# Establishment of dynamic nomogram and risk score models for T2DM: a retrospective cohort study in Beijing

**DOI:** 10.1186/s12889-022-14782-6

**Published:** 2022-12-09

**Authors:** Chao Tong, Yumei Han, Shan Zhang, Qiang Li, Jingbo Zhang, Xiuhua Guo, Lixin Tao, Deqiang Zheng, Xinghua Yang

**Affiliations:** 1 School of Public Health, Capital Medical University, NO.10 Xitoutiao, Youanmen, Beijing, 100069 China; 2 Beijing Physical Examination Center, No. 59, Beiwei Road, Xicheng District, Beijing, China

**Keywords:** Diabetes mellitus, Dynamic nomogram, Risk score, Real world study

## Abstract

**Background:**

Health interventions can delay or prevent the occurrence and development of diabetes**.** Dynamic nomogram and risk score (RS) models were developed to predict the probability of developing type 2 diabetes mellitus (T2DM) and identify high-risk groups.

**Methods:**

Participants (*n* = 44,852) from the Beijing Physical Examination Center were followed up for 11 years (2006–2017); the mean follow-up time was 4.06 ± 2.09 years. Multivariable Cox regression was conducted in the training cohort to identify risk factors associated with T2DM and develop dynamic nomogram and RS models using weighted estimators corresponding to each covariate derived from the fitted Cox regression coefficients and variance estimates, and then undergone internal validation and sensitivity analysis. The concordance index (C-index) was used to assess the accuracy and reliability of the model.

**Results:**

Of the 44,852 individuals at baseline, 2,912 were diagnosed with T2DM during the follow-up period, and the incidence density rate per 1,000 person-years was 16.00. Multivariate analysis indicated that male sex (*P* < 0.001), older age (*P* < 0.001), high body mass index (BMI, *P* < 0.05), high fasting plasma glucose (FPG, *P* < 0.001), hypertension (*P* = 0.015), dyslipidaemia (*P* < 0.001), and low serum creatinine (sCr, *P* < 0.05) at presentation were risk factors for T2DM. The dynamic nomogram achieved a high C-index of 0.909 in the training set and 0.905 in the validation set. A tenfold cross-validation estimated the area under the curve of the nomogram at 0.909 (95% confidence interval 0.897–0.920). Moreover, the dynamic nomogram and RS model exhibited acceptable discrimination and clinical usefulness in subgroup and sensitivity analyses.

**Conclusions:**

The T2DM dynamic nomogram and RS models offer clinicians and others who conduct physical examinations, respectively, simple-to-use tools to assess the risk of developing T2DM in the urban Chinese current or retired employees.

**Supplementary Information:**

The online version contains supplementary material available at 10.1186/s12889-022-14782-6.

## Background

Type 2 diabetes mellitus (T2DM) is a complex metabolic disease prevalent worldwide [1–3]. The World Health Organization estimated that, globally, 422 million adults aged over 18 years were living with diabetes in 2014 [4]. Currently, China has the largest number of patients with diabetes worldwide, and the prevalence of diabetes in China ranks among the top in the world [5, 6]. Indeed, several previous studies succeeded have proven that lifestyle interventions can be effective in the prevention of T2DM [7, 8]. However, Heidemann et al. found that some individuals perceived their diabetes risk to be low, even though the actual risk was high [9].

In recent years, numerous diabetes risk score (RS) models have been developed to predict the risk of T2DM incidence [10, 11]. However, various diabetes risk models and scores were rarely used because they were developed without a specific user or explicit use in mind [12]. Thus, several investigators have developed risk scores specifically for rural or urban population, respectively [13–15]. In addition, several diabetes RS models have been constructed for specific age groups [16, 17]. However, no study has developed an RS model specially for the current or retired employees. Moreover, based on normal nomogram, dynamic nomogram is an online scoring system that provides clinicians with a simple-to-use tool to tailor clinical decisions [18]. To our knowledge, the dynamic nomogram has not yet been developed for predicting the probability of T2DM incidence.

This study aimed to develop a dynamic nomogram and an RS model based on physical examination data to predict the probability of developing T2DM and identify high-risk populations, and to provide a basis for health management and risk communication in primary care practice.

## Methods

### Study population

We used retrospective cohort data from the Beijing Health Management Cohort (BHMC), which covers current or retired employees in Beijing, China [19]. Individuals were included in the dynamic cohort if they were older than 16 years old, had records at least three medical examinations, without T2DM at the time of entry into the cohort. The members with (a) missing data on important variables such as age, sex, FPG levels, and clinical laboratory indicators (*n* = 134) and (b) diabetes (*n* = 4536), cardiovascular and cerebrovascular diseases (*n* = 49), liver and kidney diseases (37), and family history of diabetes (*n* = 134) were excluded. According to the Chinese Guidelines for the Prevention and Treatment of T2DM (2017 edition), participants were diagnosed with diabetes and discontinued from follow up when the fasting plasma glucose (FPG) level was ≥ 7.0 mmol/L [20]. Finally, a total of 44,852 individuals were enrolled in the study population, which including 24,817 men and 20,035 women. The age of the population ranged from 16 to 68 years, with an average age of 38.48 ± 11.87 years. The project was approved by the ethics committee of the Beijing Physical Examination Center (Beijing, China; ethics approval ID: 201,802 & 202,008). All methods were carried out in accordance with relevant guidelines and regulations, and informed consent was obtained from 11,145 participants. As this was a retrospective study, the ethics committee of the Beijing Physical Examination Center approved the remaining participants with waived written informed consent.

### Measurements and surveys

Data from January 2006 to December 2017 were used in this study. The project data included anthropometric measurements (height, weight, and blood pressure [BP]), blood biochemical indicators (FPG, serum creatinine [sCr], blood uric acid [SUA], estimated glomerular filtration rate [eGFR], total cholesterol, triglycerides [TGs], low-density lipoprotein cholesterol [LDL-C], and high-density lipoprotein cholesterol [HDL-C]), and questionnaire survey (sex, age, history of DM and hypertension, and family history of diabetes). eGFR was calculated using the abbreviated Modification of Diet in Renal Disease equation:

$$\mathrm{eGFR}=175\ast\mathrm{creatinine}^{-1.154}\ast\mathrm{age}^{-0.203}\ast1.212(\mathrm{if}\;\mathrm{black})\ast0.742(\mathrm{if}\;\mathrm{female})$$

In accordance with the guidelines for prevention and control of overweight and obesity in Chinese adults, body mass index (BMI) was categorized into four groups: underweight (< 18.5 kg/m^2^), normal (18.5 to < 24.0 kg/m^2^), overweight (24.0 to < 28.0 kg/m^2^), and obesity (≥ 28.0 kg/m^2^) [21]. Hypertension was diagnosed as an average systolic BP (SBP) ≥ 140 mmHg, or diastolic BP (DBP) ≥ 90 mmHg, based on the US Seventh Joint National Committee (JNC7) [22]. Dyslipidaemia was defined as total cholesterol (TC) ≥ 6.2 mmol/L or TG ≥ 2.3 mmol/L or LDL-C ≥ 4.1, or HDL-C < 1.0 mmol/L, according to the 2016 Chinese Guideline for the Management of Dyslipidaemia in Adults [23]; Hyperuricaemia was defined as SUA ≥ 360 μmol/L in women or ≥ 420 μmol/L in men under normal purine diet [24]. eGFR was divided into two categories: eGFR ≥ 90 mL/min/1.73 m^2^ and eGFR < 90 mL/min/1.73 m^2^ [25]. sCr was transformed into categorical variables according to quartiles.

### Statistical analysis

Categorical variables were expressed as numbers (percentages) and compared using the chi-square test. For nomogram construction and internal validation, we randomly divided the study population into training (*n* = 31,391) and validation (*n* = 13,461) cohorts in a ratio of 7:3. The univariate and multivariate Cox proportional hazards models were conducted in the training cohort to develop a nomogram using weighted estimators corresponding to each covariate derived from the fitted Cox regression coefficients and variance estimates [26]. An RS was calculated by summing the risk points corresponding to each of the weighted covariates used to perform the dynamic nomogram. Individuals were classified into three groups according to the risk of developing diabetes. The dynamic nomogram was evaluated using ten-fold cross-validation. Kaplan–Meier survival curves were plotted to estimate the probability of remaining free of T2DM during the follow-up for each group of subjects in the training and validation sets. Besides, exploratory subgroup analysis was used to examine the effect of employment status on the RS model. According to the general retirement age in China, retirement was defined as age ≥ 50 years in women or ≥ 60 years in men [27].Sensitivity analysis was conducted in a cohort that excluded the first two years of follow-up to validate the accuracy of the model.

For all analyses, a two-tailed *p*-value of < 0.05 was defined as statistically significant. SPSS software (version 26.0; IBM, Chicago, United States) was used for general descriptive analysis and χ^2^-tests. Univariate and multivariate Cox proportional hazards regression models, dynamic nomogram, tenfold cross-validation, and Kaplan–Meier survival curves were performed using R statistical software (version 4.0.2; R Foundation for Statistical Computing, Vienna, Austria).

## Results

### Baseline characteristics of participants

The baseline characteristics of the study cohort are presented in Table 1. A total of 44,852 participants without diabetes at baseline were assigned into two groups based on whether they had diabetes during the follow-up period. For over a median follow-up of 3.58 years (interquartile range, 2.31–5.10 years), 2,912 (6.49%) participants developed new-onset T2DM (16.00 events per 1,000 person-years at follow-up). Individuals with new-onset diabetes during follow-up tended to be older, men and retired, have higher levels of BMI, FPG, sCr and eGFR, and were more likely to have hypertension, dyslipidaemia, and hyperuricaemia.

**Table 1:** Baseline characteristics of participants

	Overall (*n* = 44,852)	Diabetes-free (*n* = 41,940)	New diabetes (*n* = 2,912)	*P-*value
Gender (n, %)				< 0.001
Male	24,817 (55.3)	22,648 (54.0)	2,169 (74.5)	
Female	20,035 (44.7)	19,292 (46.0)	743 (25.5)	
Age, years (n, %)				< 0.001
< 30	15,067 (33.6)	14,866 (35.4)	201 (6.9)	
30–39	11,440 (25.5)	10,949 (26.1)	491 (16.9)	
40–49	9,648 (21.5)	8,731 (20.8)	917 (31.5)	
50–59	6,631 (14.8)	5,737 (13.7)	894 (30.7)	
≥ 60	2,066 (4.6)	1,657 (4.0)	409(14.0)	
Employment status				< 0.001
Employed	39,812 (88.8))	37,580 (89.6)	2,232 (76.6)	
Retired	5,040 (11.2)	4,360 (10.4)	680 (23.4)	
BMI status (n, %)				< 0.001
Underweight	2,093 (4.7)	2,084 (5.0)	9 (0.3)	
Normal	21,628 (48.2)	21,063 (50.2)	565 (19.4)	
Overweight	15,258 (34.0)	13,931 (33.2)	1,327 (45.6)	
Obesity	5,873 (13.1)	4,862 (11.6)	1.011 (34.7)	
FPG, mmol/L (n, %)				< 0.001
< 5.6	37,789 (84.3)	37,240 (88.8)	549 (18.9)	
5.6–6.1	3,381 (7.5)	2,632 (6.3)	749 (25.7)	
6.1–7.0	3,682 (8.2)	2,068 (4.9)	1,614 (55.4)	
Hypertension (n, %)				< 0.001
No	41,600 (92.7)	39,271 (93.6)	2,329 (80.0)	
Yes	3,252 (7.3)	2,669 (6.4)	583 (20.0)	
Dyslipidaemia (n, %)				
No	33,355 (74.4)	31,903 (76.1)	1,452 (49.9)	
Yes	11,497 (25.6)	10,037 (23.9)	1,460 (50.1)	
Hyperuricemia (n, %)				< 0.001
No	38.995 (86.9)	36,762 (87.7)	2,233 (76.7)	
Yes	5,857 (13.1)	5,178 (12.3)	679 (23.3)	
sCr, μmol/L (n, %)				< 0.001
< 57.2	11,178 (24.9)	10,723(25.6)	455(15.6)	
57.2–68.1	11,274 (25.1)	10,571(25.2)	703(24.1)	
68.2–78.9	11,246 (25.1)	10,397(24.8)	849(29.2)	
> 78.9	11,154 (24.9)	10,249(24.4)	905(31.1)	
eGFR, mL/min/1.73m^2^ (n, %)				< 0.001
≥ 90	33,670 (75.1)	31,786 (75.8)	1,884 (64.7)	
< 90	11,182 (24.9)	10,154 (24.2)	1,028 (35.3)	

Categorical variables are presented as numbers (percentages) and compared using the chi-square test

Abbreviations: BMI, body mass index; FPG, fasting plasma glucose; sCr, serum creatinine; eGFR, estimated glomerular filtration rate

### Independent high-risk factors associated with incidence

Further multivariate Cox regression analysis showed that male sex (HR = 1.76, *P* < 0.001), age (30–39 years) (HR = 1.54, *P* < 0.001), age (40–49 years) (HR = 1.99, *P* < 0.001), age (50–59 years) (HR = 2.45, *P* < 0.001), age (≥ 60 years) (HR = 3.41, *P* < 0.001), underweight (HR = 0.46, P = 0.029), overweight (HR = 1.45, *P* < 0.001), obesity (HR = 2.22, *P* < 0.001), FPG (5.6–-6.1 mmol/L) (HR = 9.90, *P* < 0.001), FPG (6.2–-7.0 mmol/L) (HR = 21.69, *P* < 0.001), hypertension (HR = 1.15, P = 0.015), dyslipidaemia (HR = 1.28, *P* < 0.001), sCr (57.2–-68.1 μmol/L) (HR = 0.92, P = 0.321), sCr (68.2–78.9 μmol/L) (HR = 0.68, *P* < 0.001), and sCr > 78.9 μmol/L (HR = 0.63, *P* < 0.001) were independent influential factors associated with the development of T2DM (Table 2).

**Table 2:** Univariate and multivariate Cox proportional hazards regression analysis of new-onset diabetes in the training cohort

Variable names	Univariate Cox regression model		Multivariable Cox regression model	
	HR	95%CI	HR	95%CI
Gender				
Female	1		1	
Male	2.63	2.38–2.91	1.76	1.49–2.07
Age (years old)				
< 30	1		1	
30–39	2.98	2.45–3.62	1.54	1.27–1.88
40–49	5.96	4.97–7.15	1.99	1.64–2.40
50–59	9.44	7.88–11.32	2.45	2.02–2.98
≥ 60	14.95	12.23–18.26	3.41	2.75–4.24
BMI status				
Normal	1		1	
Underweight	0.20	0.10–0.41	0.46	0.23–0.92
Overweight	3.31	2.94–3.72	1.45	1.28–1.64
Obesity	7.19	6.36–8.13	2.22	1.95–2.54
FPG (mmol/L)				
< 5.6	1		1	
5.6–6.1	15.37	13.46–17.51	9.90	8.65–11.33
6.2–7.0	39.50	35.18–44.35	21.69	19.13–24.60
Hypertension				
No	1		1	
Yes	3.47	3.12–3.86	1.15	1.03–1.28
Dyslipidaemia				
No	1		1	
Yes	3.02	2.77–3.29	1.28	1.17–1.40
Hyperuricemia				
No	1		1	
Yes	2.17	1.96–2.40	1.04	0.94–1.16
sCr (μmol/L)				
< 57.2	1		1	
57.2–68.1	1.57	1.36–1.81	0.92	0.77–1.09
68.2–78.9	1.86	1.63–2.14	0.68	0.56–0.83
> 78.9	2.05	1.79–2.34	0.63	0.48–0.82
eGFR (mL/min/1.73m^2^)				
≥ 90	1		1	
< 90	1.52	1.39–1.66	0.96	0.82–1.12

HR was calculated with female vs. male sex, age < 30 years vs. age 30–39 years or age 40–49 years or age 50–59 years or age ≥ 60 years, normal vs. overweight or obesity, FPG < 5.6 mmol/L vs. FPG 5.6–6.1 mmol/L or 6.2–7.0 mmol/L, with vs. without hypertension, with vs. without dyslipidaemia, with vs. without hyperuricaemia, sCr < 57.2 μmol/L vs. sCr 57.2–68.1 μmol/L or sCr 68.2–78.9 μmol/L, eGFR ≥ 90 mL/min/1.73 m^2^ vs. eGFR < 90 mL/min/1.73 m^2^

Abbreviations: HR, hazard ratio; CI, confidence interval; BMI, body mass index; FPG, fasting plasma glucose; sCr, serum creatinine; eGFR, estimated glomerular filtration rate

### Predictive dynamic nomogram and RS on the incidence of diabetes

**Fig. 1:**
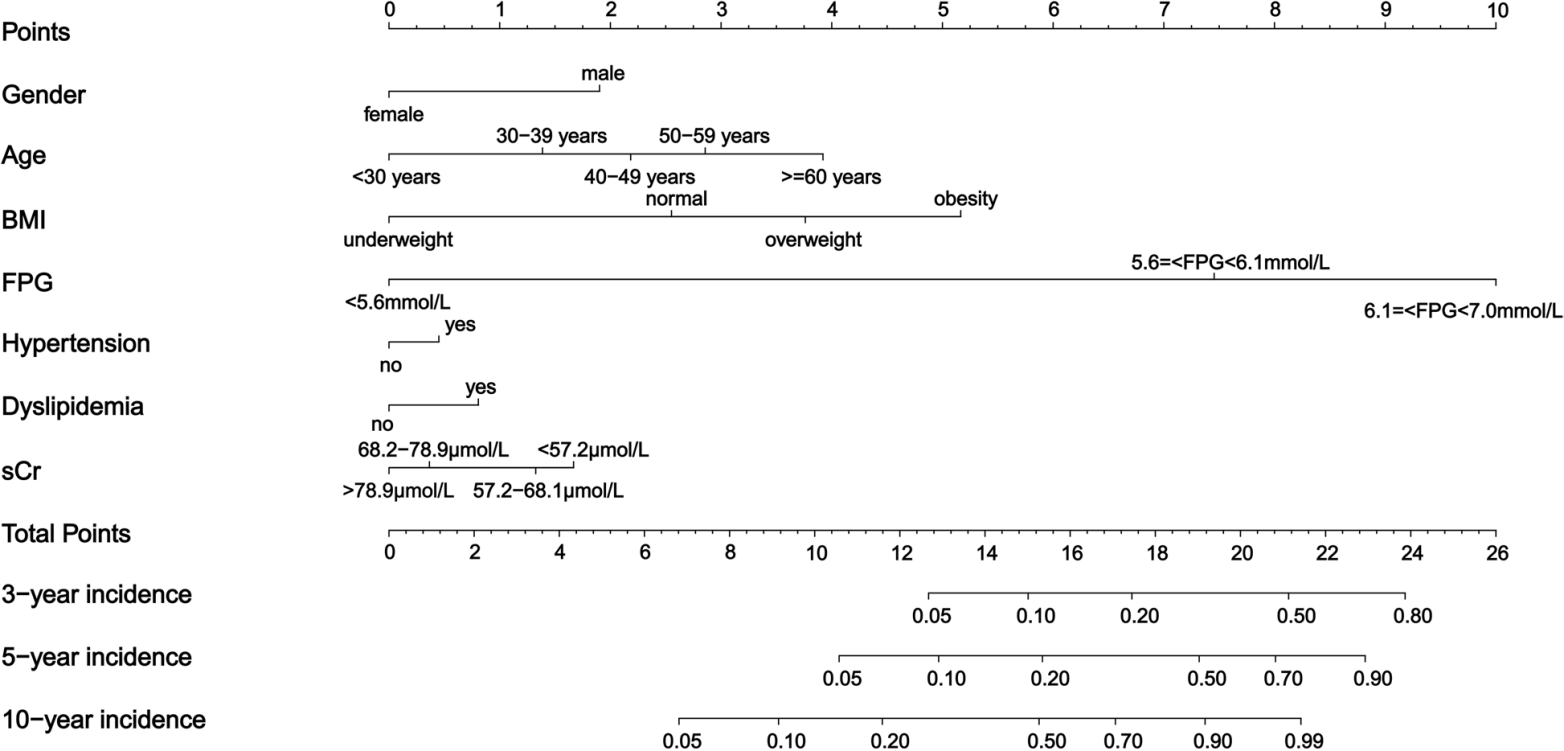
Nomogram predicting the 3-, 5- and 10-year incidence of T2DM based on the multivariable Cox proportional hazard model in the training cohort. The value of each variable was given a score on a point scale from 0 to 10. The total points projected on the bottom scales indicate the probabilities of 3-, 5-, and 10-year morbidities

Figure 1 presents a nomogram established for the 3-, 5- and 10-year incidence of diabetes based on the above multivariable Cox proportional hazard model in the training cohort. The nomogram demonstrated that FPG levels contributed the most to the incidence, followed by sex, age, BMI, dyslipidaemia, hypertension, and sCr, which had good accuracy in estimating the risk of developing diabetes, with a concordance index (C-index) of 0.909 in the training set and 0.905 in the validation set. Ten-fold cross-validation estimated the AUC of the dynamic nomogram at 0.909 (95% CI 0.897–0.920). Additionally, a diabetes prediction dynamic nomogram application (https://nomogramxhy.shinyapps.io/DynNomapp/) was produced, which can be conveniently available to clinicians in primary care. Using the application, one individual’s risk probability of remaining T2DM-free in the following years can be obtained immediately when imputing the information of the seven identified variables. The R code and data for the application were attached in Supplement Data.

Our RS was calculated based upon the weighted of diabetes risk factors as follows: (1.90 × I [male sex]) + (1.39 × I [30–39 years age]) + (2.18 × I [40–49 years age]) + (2.86 × I [50–59 years age]) + (3.92 × I [≥ 60 years age]) + (2.55 × I [normal]) + (3.76 × I [overweight]) + (5.16 × I [obesity]) + (7.45 × I [5.6 ≤ FPG < 6.1 mmol/L]) + (10.00 × I [6.1 ≤ FPG < 7.0 mmol/L]) + (0.45 × I [hypertension]) + (0.81 × I [dyslipidaemia]) + (0.37 × I [68.2–78.9 μmol/L sCr]) + (1.33 × I [57.2–68.1 μmol/L sCr]) + (1.67 × I [< 57.2 μmol/L sCr]), where I [] denotes the indicator function that is equal to 1 if the condition in the parentheses is satisfied, and 0 otherwise.

**Fig. 2:**
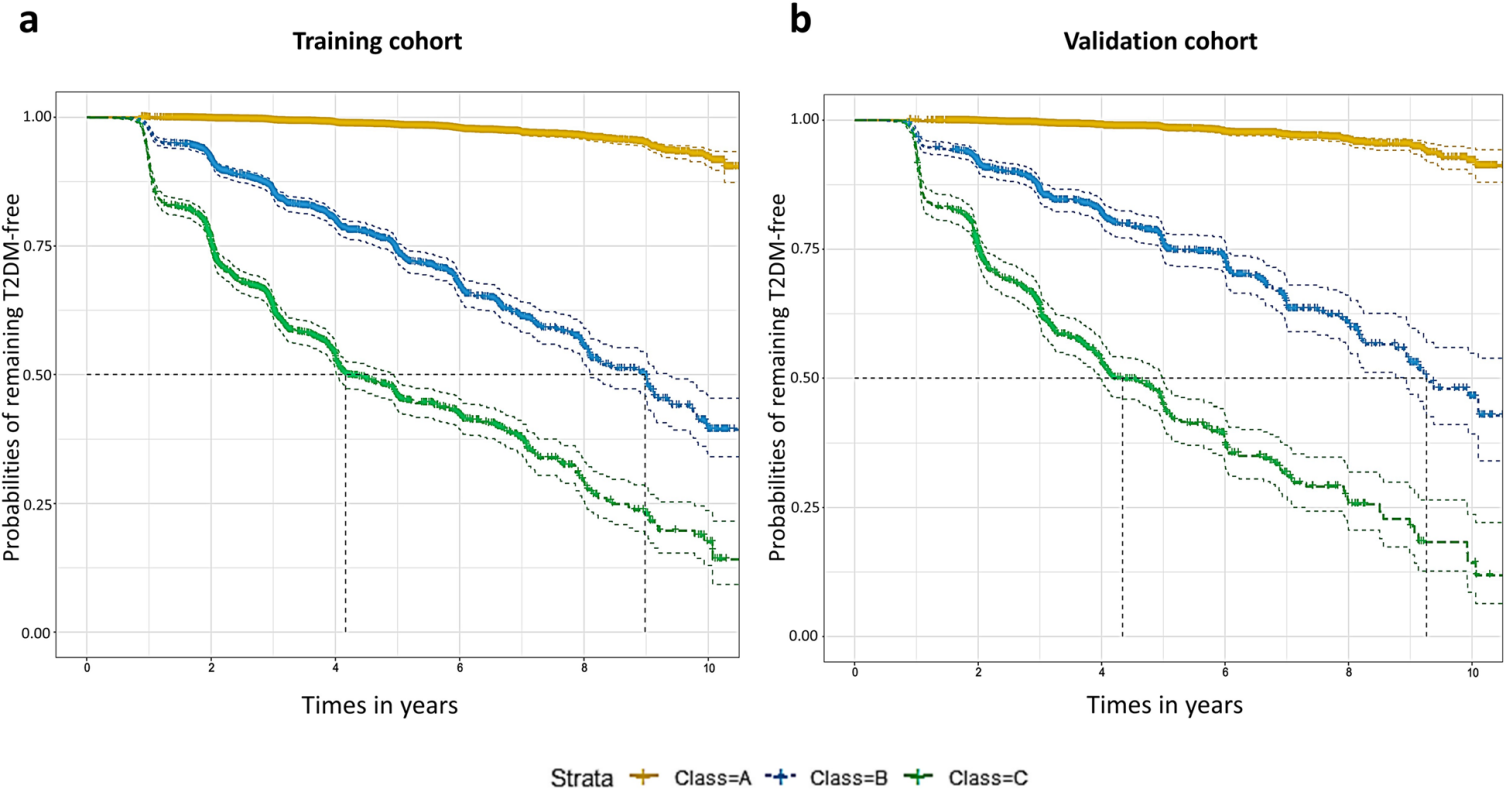
Comparison of the probabilities of remaining T2DM-free in each class according to the disease index. **a** training cohort (*n* = 31,391); **b** validation cohort (*n* = 13,461). Class A, 0–12 points in the nomogram (yellow); Class B, 12–18 points in the nomogram (blue); Class C, 18–26 points in the nomogram

Furthermore, RS was classified into three levels of risk according to the risk of developing diabetes: those with 0–12 points who had less than 10% incidence of diabetes been considered low risk (Class A), 12–18 points with 10%–50% incidence of diabetes was mid-risk (Class B), and 18–-26 points with over 50% incidence was high risk (Class C). The Kaplan–Meier survival curve for both cohorts according to RS was shown in Fig. 2. Each set of RS class appeared well separated, indicating reasonable discrimination in the cohort (*P* < 0.001). Using this predictive model, people can directly calculate their RS and categorize themselves into different risk levels and obtain a simple understanding of the risk of developing T2DM and how they may develop in the following years by reviewing the nomogram and Kaplan–Meier survival curve.

### Subgroup analysis

We conducted subgroup analysis to examine the effect of employment status on the RS model. A total of 39,812 participants were included into the employed cohort, with a median follow-up of 3.62 years (interquartile range, 2.28–5.10 years), 2,232 (5.6%) employed developed new-onset T2DM. The nomogram had good accuracy in estimating the risk of developing diabetes in the employed cohort, with a C-index of 0.910. In addition, 5,040 individuals were included into the retired cohort, with a median follow-up of 3.22 years (interquartile range, 2.61–5.08 years), 680 (13.5%) retired developed T2DM. The nomogram had good accuracy in the retired cohort, with a C-index of 0.867. The Kaplan–Meier survival curves for the employed and retired cohorts according to RS were shown in Figure S2, and each set of RS classes appeared well discriminated in the two cohorts (*P* < 0.001).

### Sensitivity analysis

We conducted sensitivity analysis by excluding the first two years of follow-up. A total of 37,694 participants were included into the medium- and long-term cohort. For over a median follow-up of 3.93 years (interquartile range, 2.95–5.69 years), 1,778 (4.6%) participants developed new-onset T2DM (10.39 events per 1,000 person-years at follow-up). The nomogram had good accuracy in estimating the risk of developing diabetes in the medium- and long-term cohort, with a C-index of 0.888. The Kaplan–Meier survival curve for the cohort according to RS was shown in Figure S1, and each set of RS classes appeared well discriminated in the cohort (*P* < 0.001).

## Discussion

The primary aim of this study was to develop a dynamic nomogram and an RS model based on physical examination data to predict the probability of T2DM incidence. Multiple Cox regression demonstrated that male sex, older age, high BMI, high FPG, hypertension, dyslipidaemia, and low sCr at presentation were risk factors for T2DM. In addition, dynamic nomogram and RS models were constructed using weighted estimators corresponding to each covariate derived from the fitted Cox regression coefficients and variance estimates. The dynamic nomogram achieved a high C-index of 0.909 in the training set and 0.905 in the validation set. A tenfold cross-validation estimated the AUC of the nomogram at 0.909 (95% CI: 0.897–0.920).

According to the International Diabetes Federation Diabetes Atlas 9th edition (2019), diabetes is one of the fastest growing health challenges in the twenty-first century, and the number of adults with diabetes has more than tripled in the past 20 years [28]. Currently, China has the largest number of diabetes cases worldwide, and this number continues to grow, putting constant strains on healthcare resources [6]. Yang et al. interviewed 1482 adults with diabetes and 1553 adults with glucose tolerance in the normal range to estimate the expenditures for medical care caused by diabetes in China [29]. The study showed that expenditures for healthcare were 3.38 times higher among people with diabetes than among those with normal glucose tolerance. Fortunately, many studies have found that lifestyle interventions, such as weight-loss, improved diet, and physical activity, substantially decreased the incidence of diabetes among high-risk individuals [30, 31]. Therefore, it is important to identify individuals at risk of developing T2DM and provide targeted health guidance.

In the current study, for over a median follow-up of 3.58 years (interquartile range, 2.31–5.10 years), 2,912 (6.49%) participants developed new-onset T2DM, which is based on the Beijing Health Management Cohort. This cohort includes current or retired employees who are representative of the urban population in China, with a good follow-up stability. According to previous studies, the prevalence of diabetes is higher in Beijing compared to the overall Chinese population [32–34], which may be due to the fact that Beijing has the highest prevalence of overweight and obesity among children and adults in China [35].

A nationwide population-based survey in Germany showed that 78.9% of high-risk individuals perceived themselves as having diabetes risk as almost absent or slight, demonstrating that providing effective risk communication is essential [9]. In the past 20 years, experts have been striving to develop weighted models that can be presented as scores to reflect the complexity of prediction models while being perceived as sufficiently simple, credible, affordable, and widely implementable in practice [10, 11]. However, Schmid et al. found that the prevalence of participants at risk for T2DM varied considerably depending on the scoring system used [36]. Most of the RS models were described as “simple” or “easily implemented”, and less were specific about target users and environments [12]. To adequately prevent T2DM, a risk scoring system should be constructed for each population. Several investigators established special RS models of T2DM for the rural Chinese population, but the AUC values for all models were less than 0.800 [14, 15, 37]. Pires de Sousa et al. developed a simple risk score for the Brazilian urban population, and the model achieved an AUC of 0.720 through external validation [13]. Furthermore, a study following a cohort of 6342 middle-aged adults developed a risk score derived from simple biochemical examination, with an AUC of the model at 0.77. To our knowledge, few diabetes risk models have been constructed based on primary healthcare. Our dynamic nomogram and RS model are simple-to-use tools for assessing the risk of developing T2DM in the current or retired employees with a high accuracy.

Meanwhile, Schulze et al. reported a diabetes RS based on age, waist circumference, height, history of hypertension, physical activity, smoking, and consumption of red meat, whole-grain bread, coffee, and alcohol to screen the individuals at high risk of developing T2DM [38]. The area under the receiver-operator characteristic (ROC) curve was 0.84 in the European Prospective Investigation into Cancer and Nutrition (EPIC) -Potsdam and 0.82 in the EPIC-Heidelberg studies. However, a systematic review suggested that the use of non-invasive diabetes risk assessment tools is limited. The barriers of these assessment tools are common in screening and clinical tests, and include interference with physician–patient interaction, lack of training, and lack of time [39]. He et al. compared the predictive accuracy of a polyexposure score (PXS), a polygenic risk score (PGS), and a clinical risk score (CRS). The PXS factors were selected from exposure variables including physiological state, environmental exposure, and self-reported behavior, the PGS was calculated from previously derived single nucleotide polymorphism (SNP) weights of > 6 million SNPs, and the CRS factors included sex, age, family history, BMI, SBP, FPG, HDL-C, and TG. The C-statistics for the PGS, PXS, and CRS models were 0.709, 0.762, and 0.839, respectively [40]. As discussed above, clinical factors play a key role in the diabetes RS model compared to self-reported behavior, environmental exposures, and genes, while self-reported behavior are limited by recall bias and reporting bias, and genetic testing is not a widely applied procedure; therefore, Our RS model was developed based on readily available clinical indicators.

Li et al. conducted a nomogram based on an urban community located in China with 687 participants and an internally validated AUC of 0.812 [41]. This study is the first to develop diabetes risk score for the urban population in China with a good accuracy, based on a large cohort. Older age, sex, BMI, FPG, hypertension and dyslipidaemia are significant predictors of T2DM incidence [42, 43]. Bao et al. conducted a multivariable Cox proportional hazards regression analysis in a general population sample from China [44]. They found that sCr concentration is inversely related to incident T2DM in both men and women, which could be an effective indicator for discovering people at high risk of diabetes. This finding was validated in the present study. Here, we derived that a T2DM dynamic nomogram and an RS based on participants’ sex, age, BMI, FPG, hypertension, dyslipidaemia, and sCr. To our knowledge, this study is the first to construct a dynamic nomogram to predict the risk of developing T2DM. The dynamic nomogram demonstrated good accuracy in estimating the risk of developing diabetes, with a C-index of 0.909 in the training set and 0.905 in the validation set.

The strength of this study was that the participants were from the BHMC, current or retired employees in fixed work environments in Beijing, which effectively improved participant compliance and the completeness of data collection, and selection bias was controlled by the cohort. Although the cohort has not collected information on the employment status of the participants, according to the general retirement age in China, retirement was defined as age ≥ 50 years in women or ≥ 60 years in men. we conducted a subgroup analysis for different employment status. In addition, sensitivity analysis was conducted in a cohort that excluded the first two years of follow-up to validate the accuracy of the model. Meanwhile, based on normal nomogram, dynamic nomogram was developed, which provides clinicians with a simple-to-use tool to tailor clinical decisions. Our study has several limitations. First, it should be noted that reporting bias in diseases exclusion stage may have limited their ability to obtain accurate risk estimates, a prospective study is needed to confirm the accuracy and reliability of the T2DM RS model in the real world. Second, other specific indicators, such as HbA1c and Homeostatic Model Assessment of Insulin Resistance (HOMA-IR), and can be added to further improve sensitivity and specificity. Thirdly, the models have not been externally validated, therefore, the model can currently be used in primary care and external validation is required for its clinical use. Finally, the cohort should be followed up, verifying the accuracy of the models.

## Conclusions

In summary, diabetes is one of the fastest growing health challenges in the twenty-first century, and health interventions can delay or prevent the occurrence and development of diabetes. Therefore, identifying high-risk groups is essential for the early initiation of health education and therapeutic interventions. The T2DM dynamic nomogram and RS model consisting of seven clinical characteristics that are routinely collected in primary healthcare offer clinicians and others who conduct physical examination, respectively, simple-to-use tools for assessing the risk of developing T2DM in health check-up population. We recommend that T2DM dynamic nomogram and RS model be used for the urban Chinese current or retired employees.

## Supplementary Information

**Additional file 1.**

**Additional file 2.**

## Data Availability

The datasets used and/or analyzed during the current study are available from the corresponding author on reasonable request.

## Abbreviations

BHMC: Beijing Health Management Cohort
BMI: Body mass index
BP: Blood pressure
CRS: Clinical risk score
C-index: Concordance index
DBP: Diastolic blood pressure
eGFR: Estimated glomerular filtration rate
FPG: Fasting plasma glucose
PGS: Polygenic risk score
HDL-C: High-density lipoprotein cholesterol
LDL-C: Low-density lipoprotein cholesterol
PXS: Polyexposure score
RS: Risk score
SBP: Systolic blood pressure
sCr: Serum creatinine
SUA: Blood uric acid
T2DM: Type 2 diabetes mellitus
TC: Total cholesterol
TGs: Triglycerides
ROC: Receiver-operator characteristic
SNP: Single nucleotide polymorphism
HOMA-IR: Homeostatic Model Assessment of Insulin Resistance
EPIC: European Prospective Investigation into Cancer and Nutrition
