# Supplementary material for: Establishment of dynamic nomogram and risk score models for T2DM: a retrospective cohort study in Beijing
Source: BMC Public Health. 2022 Dec 9;22:2306. doi: 10.1186/s12889-022-14782-6 (PMC9733342; doi:10.1186/s12889-022-14782-6)
Supplement: Supplementary file 2 — Additional file 2. [file 12889_2022_14782_MOESM2_ESM.docx]

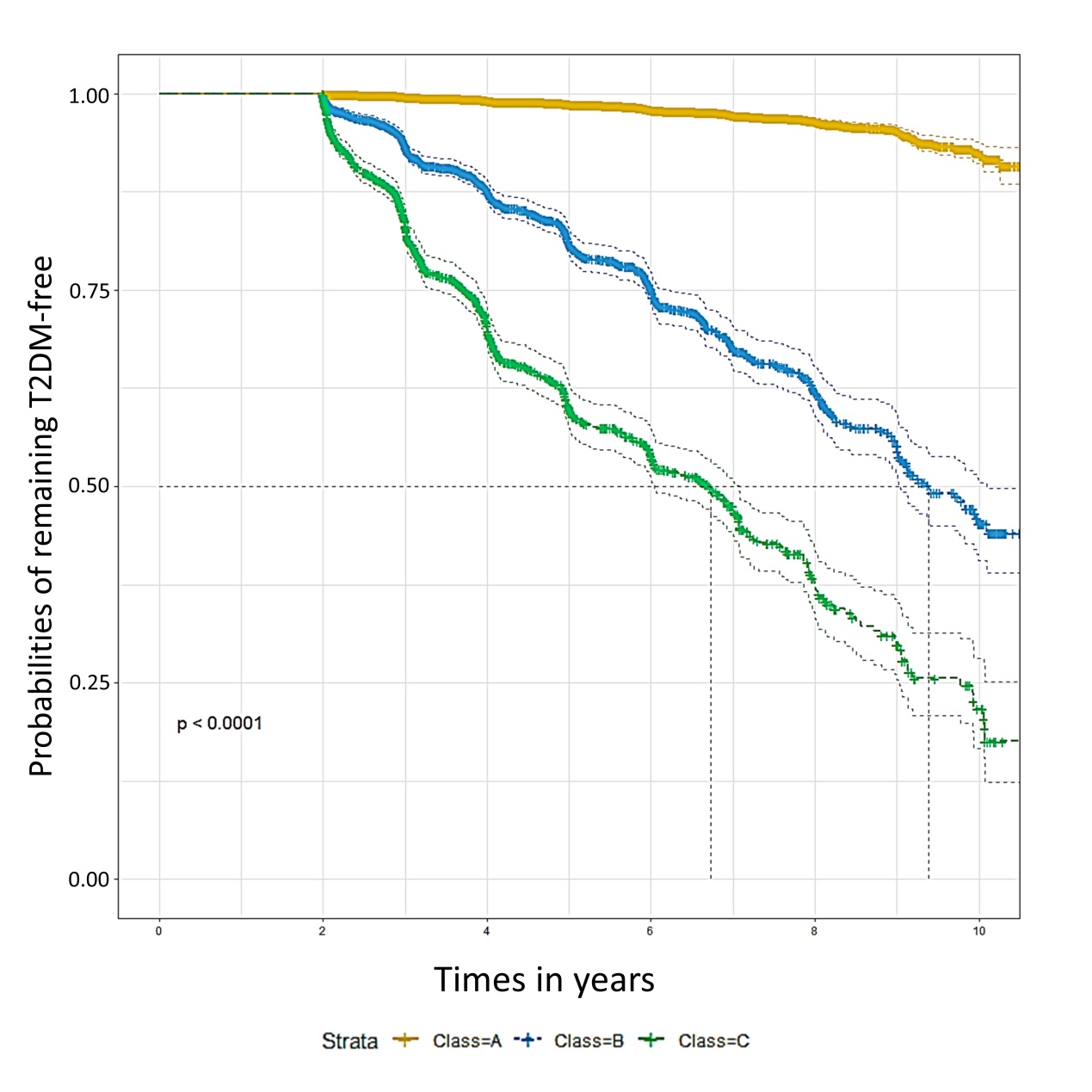


**Fig. S1** Comparison of the probabilities of remaining T2DM-free in each class according to the disease index in the medium- and long-term cohort

Class A, 0–12 points in the nomogram (yellow); Class B, 12–18 points in the nomogram (blue); Class C, 18– 26 points in the nomogram


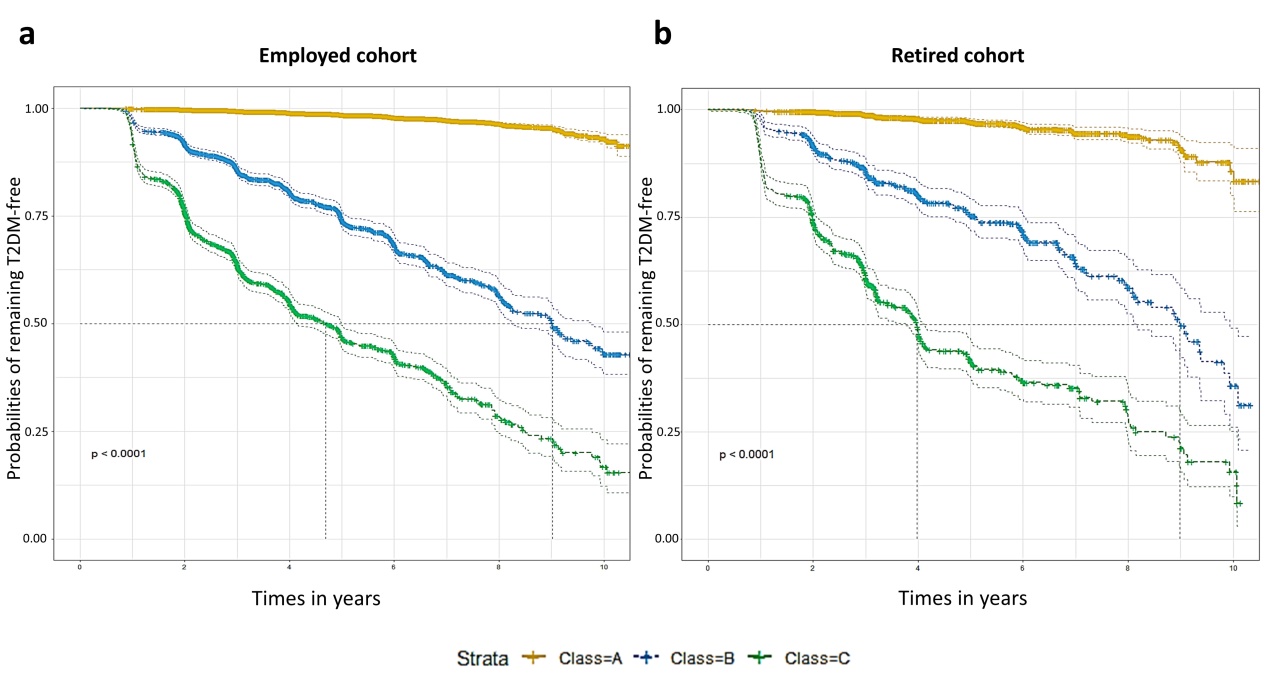


**Fig. S2** Comparison of the probabilities of remaining T2DM-free in each class according to the disease index in the employed and retired cohorts

**a** employed cohort (n = 39,812); **b** retired cohort (n = 5,040)

Class A, 0–12 points in the nomogram (yellow); Class B, 12–18 points in the nomogram (blue); Class C, 18–26 points in the nomogram
